# Design of Novel FBG-Based Sensor of Differential Pressure with Magnetic Transfer

**DOI:** 10.3390/s17020375

**Published:** 2017-02-15

**Authors:** Guohui Lyu, Guohang Che, Junqing Li, Xu Jiang, Keda Wang, Yueqiang Han, Laixu Gao

**Affiliations:** 1 Research Center for Fiber Optic Sensing Technology National Local Joint Engineering, Electronic Engineering College, Heilongjiang University, Harbin 150080, China; lvguohui@hlju.edu.cn (G.L.); 2131242@s.hlju.edu.cn (G.C.); 2151243@s.hlju.edu.cn (K.W.); 2000080@hlju.edu.cn (Y.H.); gaolaixu@hlju.edu.cn (L.G.); 2 Physics Department, Harbin Institute of Technology, Harbin 150001, China; jqli@hit.edu.cn; 3 College of Information Science and Technology, Heilongjiang University, Harbin 150080, China

**Keywords:** FBG, magnetic transmission, differential pressure sensor, beam of uniform strength

## Abstract

In this paper, a differential pressure sensor with magnetic transfer is proposed, in which the non-electric measurement based on the fiber Bragg grating (FBG) with the position limiting mechanism is implemented without the direct contact of the sensing unit with the measuring fluid. The test shows that the designed sensor is effective for measuring differential pressure in the range of 0~10 kPa with a sensitivity of 0.0112 nm/kPa, which can be used in environments with high temperature, strong corrosion and high overload measurements.

## 1. Introduction

The differential pressure sensor has been widely used in differential pressure signal measurements of fluid. When the fluid flows through the point to be measured, the tiny pressure difference between the two sampling points can be measured by the sensor [1]. Traditional electronic sensors are vulnerable to electromagnetic interference and are not suitable for applications in flammable and explosive places. In recent years, with the development of sensing technology, FBG has become one of the candidates to replace those traditional electronic sensors [2,3,4,5]. For example, different strategies have been adopted successfully in order to obtain feasible and reliable temperature sensing [6].

**Figure 1:**
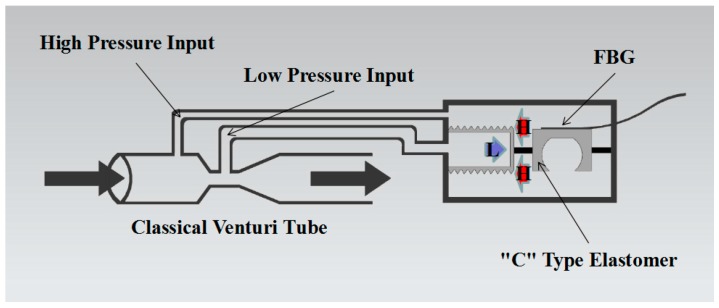
The typical structure of a differential pressure sensor in which an FBG may be used.

Figure 1 describes the typical structure of a differential pressure sensor including the FBG. In differential pressure sensing, a pressure-transferring diaphragm is required to have contact with the medium in both directions. Usually the sensing unit should be immersed in the measured fluid, which may result in interference to the measurement. In order to guarantee the sensitivity and durability of the sensor, special approaches should be taken in real measurement environments with high temperatures, high pressures, overload [7,8] and corrosion [9,10].

It should be pointed out that an FBG-sensitive element must be protected effectively, although it is extremely hard to encapsulate the FBG in a sensor in reality. In a real design, the advantage over traditional sensors and the effective protection of the FBG should be taken into account.

## 2. Designs of Differential Pressure Sensor with Magnetic Transfer

### 2.1. The Overall Design

**Figure 2:**
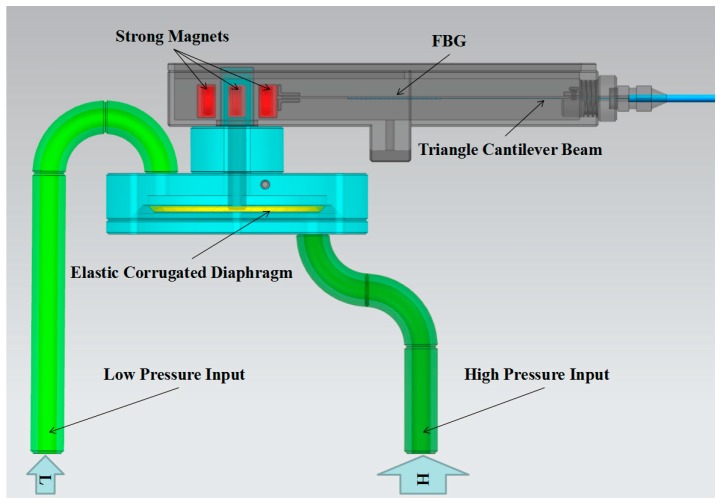
The structure of the FBG-based differential pressure sensor with magnetic transfer.

In order to sense the differential pressure effectively and to protect the sensor from invalidity caused by the contact of the FBG with the fluid to be measured, we propose a sensor structure with a magnetic transfer mechanism. As seen in Figure 2, four parts are included in this sensor structure as follows: the pressure-transferring part comprised of three strong magnets and an elastic corrugated diaphragm; the input part for differential pressure with a high pressure input channel and a low pressure input channel; the part in which a triangle cantilever beam is used to convert the differential pressure to the wavelength shift of the FBG; and a limiting mechanism which is used for resisting overload [11]. A structure design for insulating the fluid and FBG is the most critical aspect.

### 2.2. Sensing Principle of a Cantilever Beam of Uniform Strength

**Figure 3:**
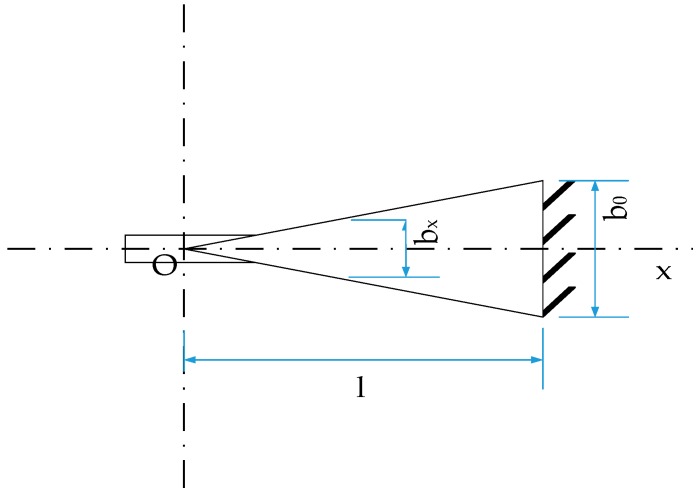
Structure of the triangle cantilever beam.

Figure 3 presents a structure of a triangle cantilever beam as an elastic sensitive element with the right end fixed and the other end movable, on which an FBG is fixed.

As shown in Figure 3, when a force *F* acts on the point O near the free end, which is just the top point of the triangle and located at a distance of *l* from the fixed end of the beam, the deformation ε of the beam caused by the force can be expressed as:

(1) $$\varepsilon (x)=\frac{6\left(l-x\right)}{E\cdot h\cdot A}\cdot F$$

where *E*, *h* and *A* indicate the elastic modulus, thickness of the beam and transverse section area, which is a product of the width *b_x_* and thickness *h* of a certain position *x* with respect to O, the top point of triangle. From Equation (1), the deformation is a function of x.

Therefore, *A* can be expressed as:

(2) $$A(x)=h\cdot b_{x}=h\cdot b_{0}\cdot \frac{l-x}{l}$$

where *b*_0_ denotes the beam width at the fixed end, *l* means the height of the triangle. Substituting Equation (2) into Equation (1), one can obtain:

(3) $$\varepsilon =\frac{6\cdot l}{E\cdot b_{0}\cdot h^{2}}\cdot F$$

From Equation (3), the deformation is equal everywhere when the point O at the movable end of the beam is applied a force *F*; therefore, the requirement for the position to fix the FBG is released. It should be pointed out that the force *F* must be applied on the point O which is the intersection between the two hypotenuses of the triangle.

The deflection *D* of the movable end of the beam is [12]

(4) $$D=\frac{6\cdot l^{3}}{E\cdot b_{0}\cdot h^{3}}\cdot F$$

From the Bragg conditions we have:

(5) $$\lambda_{B}=2n_{eff}\Lambda$$

where $n_{eff}$, Λ represent the effective refractive index and grating period of the FBG. The Bragg wavelength changes with the effective refractive index and the grating period. When a force is applied to the movable end of the beam, the FBG pasted on the surface of the beam may convert the deformation of the beam to the shift of the central wavelength of the FBG. After controlling the temperature or compensating for the effect of the temperature on the FBG (the temperature compensation design of the sensor will be introduced in a later section), the relationship between the deformation and the wavelength of the FBG can be simplified as:

(6) $$\Delta \lambda_{B}=K_{\varepsilon }\cdot \varepsilon$$

where *K_ε_* is a coefficient. Combining Equations (3) and (4) into Equation (6), we obtain:

(7) $$D=\frac{\Delta \lambda_{B}\cdot l^{2}}{K_{\varepsilon }\cdot h}$$

As seen in Equation (7), the deflection of the movable beam end *D* is proportional to the wavelength shift $\Delta \lambda_{B}$ of the FBG.

### 2.3. Structure Design of Magnetic Transfer

**Figure 4:**
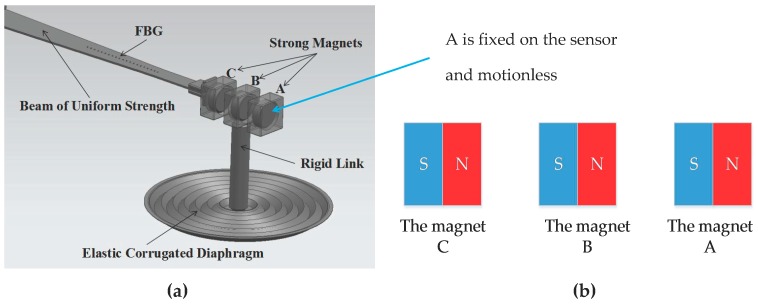
(**a**) The structure of sensitive unit with magnetic transfer; (**b**) the relationship of magnets A, B and C.

Usually, it is not easy to separate the FBG from the measured fluid. In order to solve this problem, we introduced a special mechanism with magnetic transfer to isolate the FBG from the measured fluid, where three identical strong magnets are used, described as A, B and C, respectively, in Figure 4a. Magnet A is fixed, and the S pole of A faces the N pole of B. While magnets B and C are movable, magnet B is linked rigidly with the center of the elastic corrugated diaphragm; the S pole of B faces the N pole of C, and magnet C is fixed to the movable end of the beam. A, B and C are attracted to each other and must keep equal distances of about 3~5 mm between each other. Actually, among them there must be an isolating structure (such as a polymer spacer) to prevent them from close contact. The FBG is fixed on a beam of uniform strength. It is insulated between the top and bottom surfaces of the diaphragm. When there is a difference in the pressure between the top and bottom surfaces of the diaphragm, the center of the diaphragm moves up or down, leading the movement of magnet B through the rigid link, therefore synchronously driving magnet C by means of the magnetic force. The side of the S pole of magnet C is connected to the movable end of the cantilever beam; therefore, the difference of the pressure is transferred to the FBG.

### 2.4. The Position Limiting Mechanism

**Figure 5:**
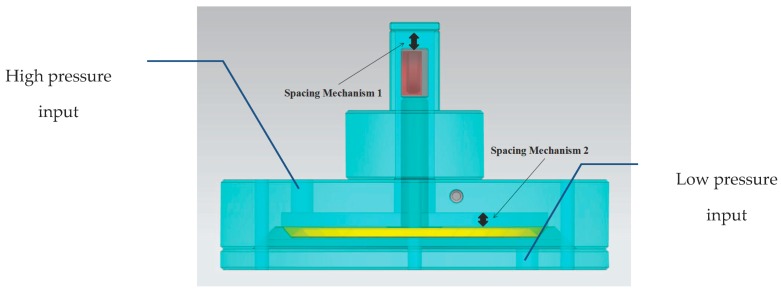
The position limiting mechanism of the sensor.

In the real design, we enabled the sensor to resist overload by introducing a position limiting mechanism [13]. In our structure, two points are needed in the careful design, as seen in Figure 5; one is for magnet B to move and the other is for the elastic corrugated diaphragm. The two points are limited to prevent the damage of the diaphragm and magnet and to avoid the invalidity of the sensor.

### 2.5. The Temperature Compensation

When a force is applied to the beam, its upper surface experiences a stretching, corresponding negative deformation, while the lower surface is compressed and experiences positive deformation, but the deformation of the upper and lower surfaces is equal in absolute value. Therefore, when two FBGs with the same temperature sensitivity coefficient are pasted on the upper and lower surfaces of the beam, respectively, the wavelength shifts of the two gratings change to the opposite direction with an identical value of the shifts. It should be pointed out that two fiber gratings are located in the same temperature environment, and the difference between the shifts of two gratings will be kept unchanged, although the change of the temperature may cause an individual shift for each grating. That means the temperature effect on the grating is eliminated and the grating gets a compensation in temperature.

## 3. Simulations of Pressure Corrugated Diaphragm and Test of Sensor

### 3.1. The Simulations of the Elastic Corrugated Diaphragm

To obtain the optimal parameters of the diaphragm and to verify the consistency of the diaphragm design with the actual case, we adopted ANSYS 14.0 (ANSYS Inc., Pittsburgh, PA, USA) to simulate the pressure diaphragm. ANSYS is a general analysis software based on the finite element method which allows us to carry out the study of the structure with heat, sound, fluid and electromagnetic fields.

**Figure 6:**
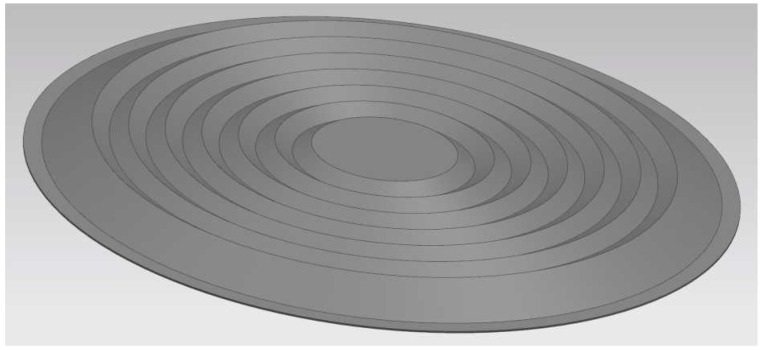
The 1:1 scale 3D model of the elastic corrugated diaphragm.

The 1:1-scale three-dimensional (3D) model of the elastic corrugated diaphragm is imported into ANSYS at first, as shown in Figure 6. In this figure, the diameter of the full-size diaphragm is 100 mm, and the corrugation type is chosen as “triangle waveform” (6 rings) with a corrugation depth of 2 mm. The material of the diaphragm is stainless steel film. And the parameters of the diaphragm are listed in Table 1.

**Figure 7:**
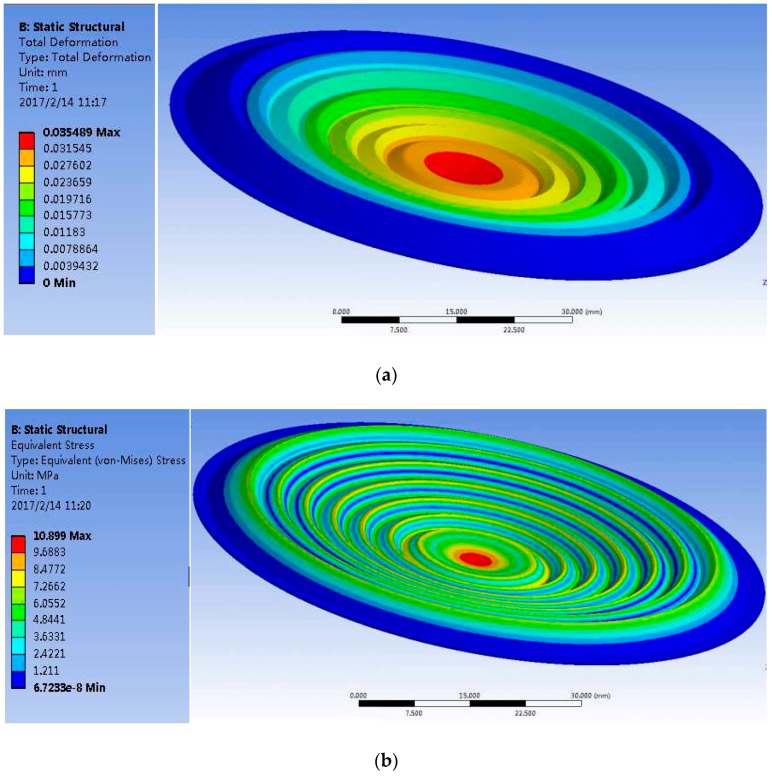
(**a**) Deformation of diaphragm; (**b**) Force distribution of diaphragm.

Then we simulated its mechanical property in a certain range of pressure differences (0~10 kPa). As an example, Figure 7 gives the deformation and force distribution of the diaphragm under a differential pressure of 10 kPa when the thickness is 0.2 mm, where the pressure difference may be reflected through stress at the center of the diaphragm.

In the real design, the edge of a diaphragm of 2 mm is reserved in advance to link with the other part of the sensor and to isolate the fluids at both sides of the diaphragm. That means the force distribution at the edge is kept unchanged.

In order to get the best diaphragm thickness, we simulated diaphragms with thicknesses of 0.2 mm, 0.1 mm, 0.05 mm, 0.025 mm, and 0.0125 mm, respectively. The simulation results are listed in Table 2.

**Figure 8:**
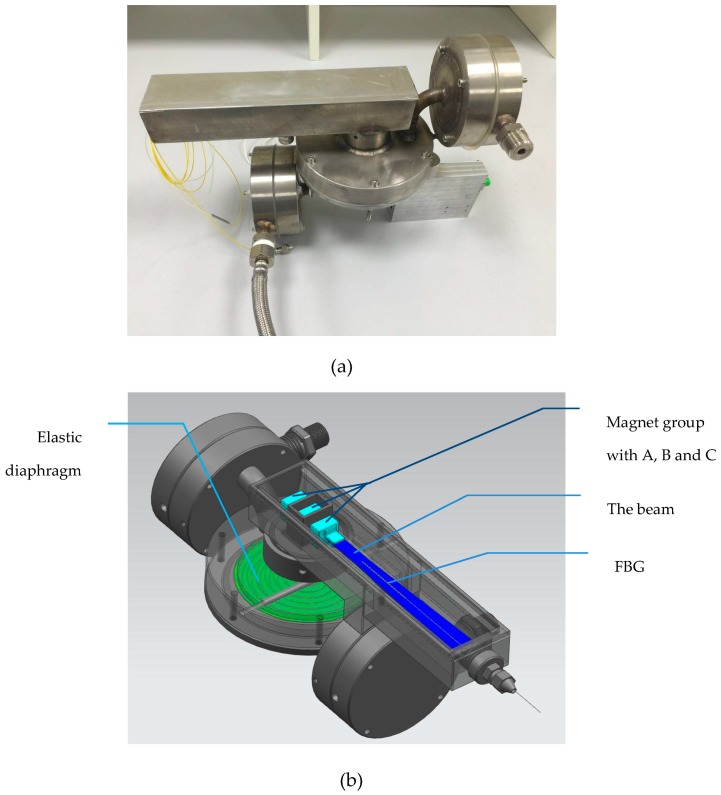
(**a**) The real sensor; (**b**) The structure inside the sensor.

The simulation results indicate a tradeoff between the thickness of the diaphragm and the measurement range. When the diaphragm is thick, the sensitivity of the diaphragm is low, which leads to an enlarged range of measurement with a lower resolution. When the diaphragm is too thin, despite the enhanced sensitivity, the measurement range is reduced and results in a degradation in endurance to the impact of a high pressure difference, even resulting in damage to the diaphragm. According to the measurement range and resolution, we chose a diaphragm that is 0.15 mm thick to fabricate and conduct tests. Figure 8 presents the real picture of the sensor.

### 3.2. Test System of the Sensor

**Figure 9:**
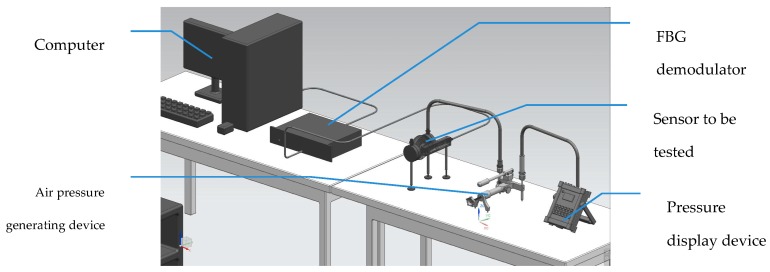
The test system of sensor.

A test system was used to evaluate the linearity of the sensor, as shown in Figure 9.

The test system included the air pressure difference–generating device, the air pressure display device, a computer, an FBG demodulator and the sensor to be tested. The air pressure–generating device was made of stainless steel, which can provide an accurate and stable pressure source. The sensor to be tested was mounted on an interface of the pressure source, and the calibrated pressure meter was mounted on another interface of the source. The value of the calibrated pressure meter was displayed as a reference value. The light signal from the sensor was analyzed by the demodulator and displayed on the PC.

Two independent pressure-generating devices are supposed to be used generally: one produces high pressure and the other produces low pressure at the same time. However, in this test, we only used one generator to provide high pressure, while the low pressure was fixed to simplify the test system. It was easy to generate differential pressure ranging from 0 to 10 kPa.

## 4. Results

**Figure 10:**
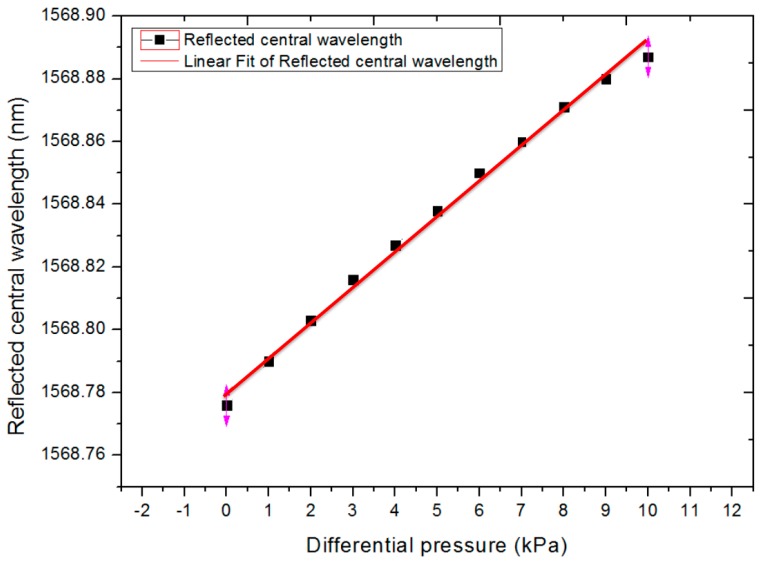
The differential pressure sensor linearity curve.

The test results (the calibration curve) are shown in Figure 10. We have fitted the data to a straight line with *λ_B_* = 0.0112 × *P* + 1568.780, with a linear correlation coefficient of 0.994, and we obtained the sensitivity of the sensor as 0.0112 nm/kPa. The relations between the central wavelength shift of the FBG and the differential pressure were almost linear.

## 5. Conclusions

In this paper, a differential pressure sensor with magnetic transfer was proposed, and a non-electric measurement method based on an FBG was introduced, which can avoid the direct contact of the sensing unit with the measuring fluid. In this design, the position limiting mechanism and temperature compensation design were also considered. The test results demonstrated that the sensor is effective to measure differential pressure in the range of 0~10 kPa with a sensitivity of 0.0112 nm/kPa, and it can operate in high temperature, strong corrosion and high overload conditions.

## Figures and Tables

**Table 1:** Parameters of the diaphragm.

Parameters	Value
Diameter of full size	100 mm
Edge width	2 mm
Number of corrugation	6
Depth of corrugation	2 mm
Spacing of corrugation	6 mm
material	stainless steel

**Table 2:** The simulation results.

Diaphragm Thickness	Maximum Deformation of Diaphragm	Maximum Pressure on the Center of Diaphragm
0.200 mm	0.035 mm	10.899 MPa
0.100 mm	0.094 mm	26.495 MPa
0.050 mm	0.172 mm	35.854 MPa
0.025 mm	0.300 mm	69.720 MPa
0.0125 mm	0.300 mm	136.830 MPa
